# Exploring the potential of machine learning for simulations of urban ozone variability

**DOI:** 10.1038/s41598-021-01824-z

**Published:** 2021-11-18

**Authors:** Narendra Ojha, Imran Girach, Kiran Sharma, Amit Sharma, Narendra Singh, Sachin S. Gunthe

**Affiliations:** 1 Physical Research Laboratory, Ahmedabad, India; 2 Space Physics Laboratory, Vikram Sarabhai Space Centre, Thiruvananthapuram, India; 3 Graphic Era (Deemed to be University), Dehradun, India; 4 Department of Civil and Infrastructure Engineering, Indian Institute of Technology Jodhpur, Jodhpur, India; 5 Aryabhatta Research Institute of Observational Sciences, Nainital, India; 6 EWRE Division, Department of Civil Engineering, Indian Institute of Technology Madras, Chennai, India; 7 Laboratory for Atmospheric and Climate Sciences, Indian Institute of Technology Madras, Chennai, India

**Keywords:** Environmental sciences, Atmospheric chemistry

## Abstract

Machine learning (ML) has emerged as a powerful technique in the Earth system science, nevertheless, its potential to model complex atmospheric chemistry remains largely unexplored. Here, we applied ML to simulate the variability in urban ozone (O_3_) over Doon valley of the Himalaya. The ML model, trained with past variations in O_3_ and meteorological conditions, successfully reproduced the independent O_3_ data (r^2^ ~ 0.7). Model performance is found to be similar when the variation in major precursors (CO and NO_x_) were included in the model, instead of the meteorology. Further the inclusion of both precursors and meteorology improved the performance significantly (r^2^ = 0.86) and the model could also capture the outliers, which are crucial for air quality assessments. We suggest that in absence of high-resolution measurements, ML modeling has profound implications for unraveling the feedback between pollution and meteorology in the fragile Himalayan ecosystem.

## Introduction

The chemical processes in the urban atmospheres of Himalayan foothills have strong potential to impact the regional air quality, agriculture, and therefore the economy^1–3^. In addition, the build-up of climate-forcing pollution in the Himalayan region can have irreversible effects on the hydrological cycle and global climate^1,2,4–7^. The atmospheric dynamics above the Himalaya also form the crossroad of so called “Atmospheric Brown Clouds” to the Tibetan Plateau^8^. Recent increase in extreme weather events triggering the calamities also indicate an intensifying interplay between the increasing pollution and meteorology over fragile ecosystem of the Himalaya^9–12^.

The enhanced concentrations of ozone (O_3_) and other climate-forcing pollutants in the Himalayan foothills are attributed to unprecedented growth in population and urbanization^13–16^. The intense forest-fires, diverse natural factors, and the topography also play vital roles in the build-up of trace gases and aerosols here^5,11,15,17–19^. The Himalayan atmosphere is particularly influenced by the most densely populated region of the world—the Indo-Gangetic Plain (IGP)^20,21^. The IGP is a global hotspot of elevated O_3_ and aerosol loading due to strong anthropogenic emissions and intense crop-residue burning in prevalence of favorable meteorological conditions^22–27^. The emissions and photochemistry in the IGP affect the Himalayan atmosphere in particular through the mountain meteorology and boundary layer dynamics^20,21,28^. A potential climate warming combined with future increase in the emissions can further intensify the atmospheric chemistry over this part of the world^29–31^.

Considering the discussed scenario, measurements and modeling studies have been conducted to assess the effects of diverse emissions, photochemistry, and dynamics on atmospheric composition over the Himalayan region^8,15,17,32,33^. The concentrations of O_3_ and precursors were found to be enhanced during pre-monsoon (spring) and post-monsoon (autumn) seasons due to regional pollution supplemented with biomass-burning, intense solar radiation, and less precipitation^15,20,21,34,35^. The long-term measurements of atmospheric composition and meteorological parameters however remain lacking over the Himalayan foothills in India, which are experiencing severe air quality and extreme weather events. Studies to fill this gap are of paramount significance since the chemistry-climate models also have greater biases in reproducing already sparse measurements over the Himalayan region^20,34,36,37^. The stronger biases are suggested to be mainly due to the limitation of models in resolving the highly complex topography of Himalaya and foothills^5,19,20,37,38^. The uncertainties in the emission inventories and parameterizations of physical and chemical processes also increase the biases in the models^19,37,39–41^. Besides higher biases, the conventional models also need intensive computing resources which poses further limitation in conducting high-resolution simulation.

In the current era, the artificial intelligence (AI) and machine learning (ML) have emerged as powerful alternative tools for modeling in various fields including the Earth system science^42–45^. Recent studies utilized AI/ML modeling in the analyses of extreme whether events and prediction of oceanic phenomenon as well as atmospheric composition^46–48^. These studies have shown that ML models trained with data from observations or physical models can produce reliable simulations without intensive high-end computing. Nevertheless, the applications of AI/ML to simulate complex atmospheric chemistry remain still limited. Considering the scientific and societal implications, lack of measurements, and limitations of conventional models over Himalayan region, the objectives of this study are as follows:

1. To explore the potential of ML modeling for simulating urban O_3_ variability.
2. To study the effects of meteorological and chemical variables on model performance.
3. To assess the effect of the data fraction used in the training on model performance.

The study region, datasets, and modeling are described in the “Methodology” section. Model simulations and results are presented in the “Model simulations and results” section, followed by “Discussion” section.

## Methodology

### Study region and datasets

The study is focussed on the urban O_3_ chemistry over the Doon valley of the Himalaya. We initiated in situ O_3_ measurements using an online O_3_ analyser manufactured by Environnement S.A, France (model O_3_ 42) at a representative station—Graphic Era deemed to be University campus (77.99° E, 30.27° N, 600 m above mean sea level). The observations are based on the UV light absorption by O_3_ and instrumental uncertainty is about 5%^49^. The continuous measurements are being conducted since April 2018 except during February–August 2020 when the laboratory was not open due to severe impact of the COVID-19 pandemic. Further details of these O_3_ measurements are presented in the earlier study^15^.

Auxiliary datasets used in training the ML model include the meteorological and chemical reanalysis from the ECMWF (European Center for Medium range Weather Forecasting). The meteorological parameters: temperature, humidity, horizontal winds, and boundary layer height (BLH) are included from the ERA-Interim^50^. Whereas, the chemical species: O_3_ and key precursors (CO, NO, NO_2_) have been included from the CAMS (Copernicus Atmosphere Monitoring Service) reanalysis^51^. ERA-Interim and CAMS products have been analyzed for diverse studies including over the Indian region^15,52–54^. The CAMS data has been shown to reproduce the day-to-day variability in the noontime O_3_ over the study region^15^. Here, we have utilized the reanalysis data of 2003–2019 period focusing on the noontime variations (6 GMT; 11:30 local time), when the urban O_3_ photochemistry is most intense.

### Machine learning model

**Figure 1:**
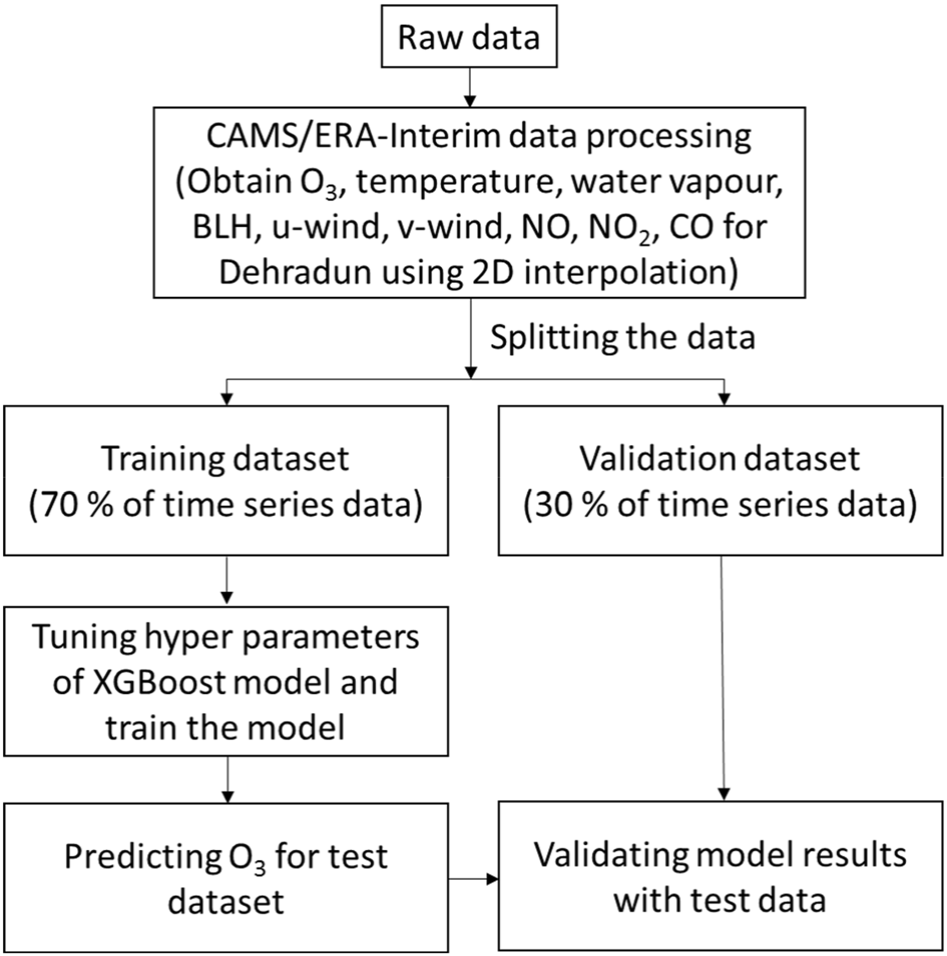
Flow chart of the steps in building the ML model for simulation of urban O_3_ variations and evaluation.

This study utilizes the XGBoost (Extreme Gradient Boosting) algorithm of the ML modeling^55^ to simulate the O_3_ variations. Considering the dependence of O_3_ on meteorological parameters and precursor gases, this modeling is under the supervised learning of AI. In the gradient boosting algorithm, a prediction model is developed in form of an ensemble of weak prediction systems i.e., decision trees. The model is built in a stage-wise manner and generalizations are made by allowing optimization of an arbitrary differentiable loss function (e.g., squared error). Further details of the XGBoost can be found elsewhere^55^. The method adopted to build and evaluate the model is shown as a flow chart in the Fig. 1.

Hyper parameters have been varied iteratively following the trial and error method to achieve better prediction. The parameters were fine-tuned using the grid search function (https://scikit-learn.org/stable/modules/generated/sklearn.model_selection.GridSearchCV.html). The values of hyper parameters set in the model are given in the supplementary material—Table S1. Other hyper parameters were kept to their default values (https://xgboost.readthedocs.io/en/latest/parameter.html). To avoid overfitting, the iterations are aborted once they cease to improve the fit parameters further, i.e., no reduction in RMSE (root mean square error) over 100 iterations. The model performance in simulating O_3_ variations has been evaluated by estimating correlation (r^2^), slope of linear fit, and RMSE (root mean square error).

## Model simulations and results

A series of simulations have been performed under this study, as summarized in the Table 1. These simulations and the evaluation of model performance are discussed in the following subsections.

**Table 1:** Different ML model simulations performed in the study.

Serial no	Simulation	Training data		
		O_3_	Meteorology	Precursors
1	ML_obs_O_3__met_prec	Measurements	ERA-interim	CAMS
2	ML_cams_O_3_	CAMS	–	–
3	ML_cams_O_3__met	CAMS	ERA-interim	–
4	ML_cams_O_3__prec	CAMS	–	CAMS
5	ML_cams_O_3__met_prec	CAMS	ERA-interim	CAMS

### Simulation utilizing in-situ O_3_ measurements

**Figure 2:**
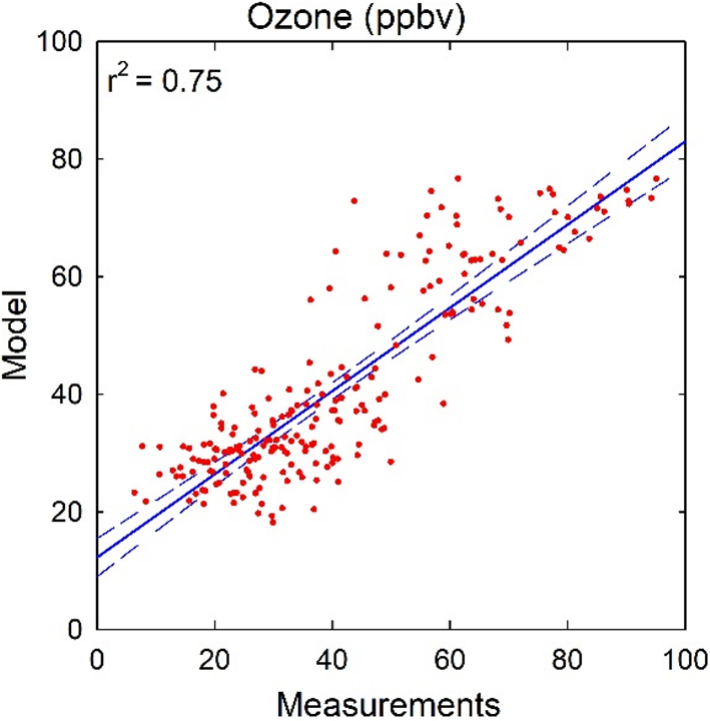
Correlation between measurements and model (ML_obs_O_3__met_prec) simulated variations in noontime O_3_ over Doon valley during April–December 2019. Solid blue line shows the linear regression fit and dashed lines show the 99% confidence intervals.

In the first simulation ML_obs_O_3__met_prec, the ML model has been trained using the observational data of O_3_ and reanalysis data of meteorological parameters (met) and precursors (prec). Analysis is focussed on the variations in noontime (11:30 h local time) O_3_. The data of April 2018 to April 2019 (number of days N = 222) has been used for training the ML model, which is 50% of total available data. Model simulation is evaluated against remaining independent observations for April–December 2019 period (N = 223 days). Figure 2 shows the correlation between the ML model simulation and in-situ measurements of noontime O_3_ over Doon valley for April–December 2019 period. ML model is found to successfully reproduce the temporal variability in the noontime O_3_ with r^2^ value of 0.75 (*p* < 0.01) and RMSE value of 10 ppbv. The estimated bias in ML model result is seen to be significantly lower as compared to the bias values reported in global and regional atmospheric models over this region^15,34,37^. The result suggests that in absence of high-resolution measurements, the ML modeling can be combined with reanalysis and limited in-situ data over the Himalayan region. The strong correlation between the ML model and in-situ measurements further opens up the possibility to utilize such simulations for assessing the impacts of O_3_ on agriculture and health in this region.

### Simulations utilizing long-term CAMS O_3_

**Figure 3:**
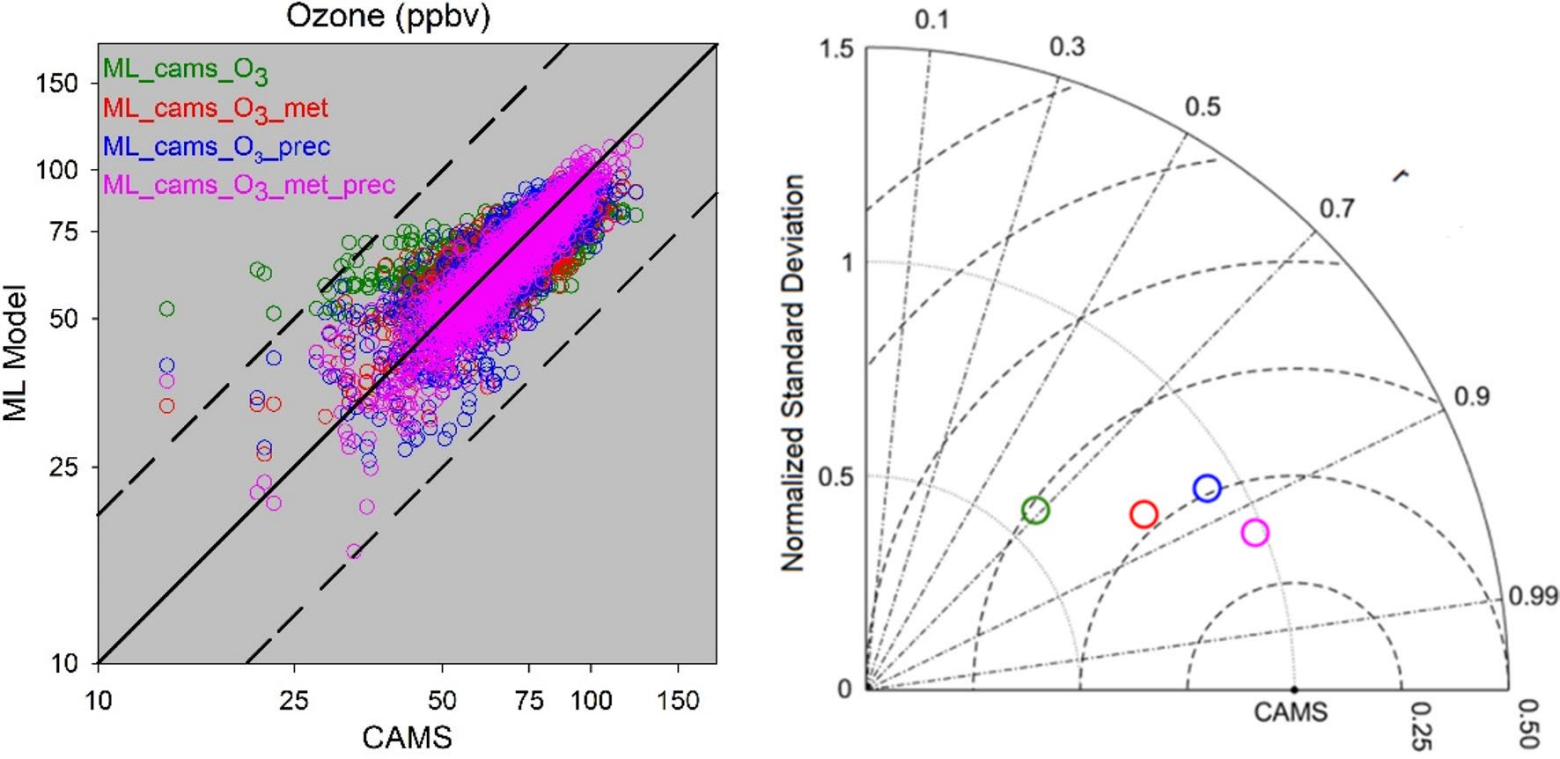
Scatter plot and Taylor′s diagram evaluating the ML model simulations of noontime O_3_ variations as compared to the CAMS reanalysis.

The in-situ measurements are largely unavailable in the Indian Himalayan region and the temporal coverage is also very limited. In view of this, we include the long-term CAMS data to assess the potential and performance of ML modeling more deeply. With availability of long-term data, here, we train the ML Model with noontime (11:30 local time) CAMS O_3_ and reanalysis meteorology for 2003–2015 (70% of total data). This makes a significant fraction (30%) of total data during 2015–2019 period available for the evaluation. The simplest simulation is ML_cams_O_3_ in which model is trained only with the O_3_ time series without including any additional parameter. This simulation is found to predict the independent O_3_ variations with r^2^ value of 0.47 and RMSE of 11.6 ppbv (Fig. 3). This result is a manifestation of a periodicity in O_3_ data embedded by the seasonal cycle in India.

The relative effect of including variations in the meteorological parameters versus major precursors (CO, NO, NO_2_) has been evaluated by performing additional simulations (Table 1, Fig. 3). Model trained with O_3_ and meteorology (ML_cams_O_3__met) reproduces independent O_3_ variations with r^2^ value of 0.71 and slope value of 0.65. Another simulation in which the ML model is trained with O_3_ and precursors but not with the meteorology shows similar or slightly improved performance (r^2^ = 0.74, slope = 0.79, *p* < 0.01). The inter-comparison of these two simulations suggests that reasonable predictions of urban O_3_ variability can be made with ML models trained with either of the meteorological or precursor dataset. This is important as this region lacks comprehensive datasets especially of the precursors, and in such cases the meteorological datasets can be used to predict O_3_. Further, to explore the potential of ML approach, we performed another simulation ML_cams_O_3__met_prec in which both meteorology as well as precursors have been included in the model. This led to significant improvement in the model performance with r^2^ value as high as 0.86 and slope value of 0.91. For this simulation, the RMSE value also drops drastically to 6 ppbv and the mean bias is also smaller (~ 3 ppbv). An important finding is that when the potentials of both meteorological as well as chemical datasets are combined, the model’s ability to predict outliers improves drastically, which is of major significance in air quality assessments.

A comparison of r^2^ values among all these simulations (numbered 2–5 in the Table 1) suggests that ~ 47% of O_3_ variations can be explained (r^2^ = 0.47 in ML_cams_O_3_) by the periodicities embedded in the data originated from the seasonal cycle. As precursors and meteorology act in tandem, higher r^2^ values (~ 0.7) in simulations trained with either meteorology or precursors suggest that this additional ~ 25% of O_3_ variability can be attributed to the changes in meteorology or precursor levels. Meteorology plus major precursors could explain ~ 86% (r^2^ = 0.86 in ML_cams_O_3__met_prec) of the variations in the urban O_3_. The remaining variability could be due to diverse unaccounted factors such as deposition, vertical transport, and volatile organic compounds, etc. The analysis suggests that ML simulations can provide deep insights into the relative importance of the physical and chemical processes affecting the air quality.

The performance of different simulations has been compiled in form of a Taylor’s diagram (Fig. 3). The figure includes statistics like r, normalized RMSE, and normalized standard deviation (SD) where normalization is done with respect to the SD in the reference (CAMS). The relative performance of different simulations is assessed by comparing how close a simulation is to the reference point (CAMS). For an ideal agreement, ML simulation should coincide with the reference point (r = 1, normalized SD = 1, and normalized RMSE = 0). It is evident that the ML simulation exploiting the potentials of both meteorology and precursors (ML_cams_O_3__met_prec) performed the best. Besides stronger r value, a normalized SD value close to 1 suggests that the simulation produces similar extent of the variability as in the CAMS. On the other hand, ML simulations using either meteorology or precursors had similar performance. Also, ML_cams_O_3__prec produced more variability likely due to non-linearities in chemistry as compared with the simulation using meteorological variations (ML_cams_O_3__met).

#### Effect of training data length

**Figure 4:**
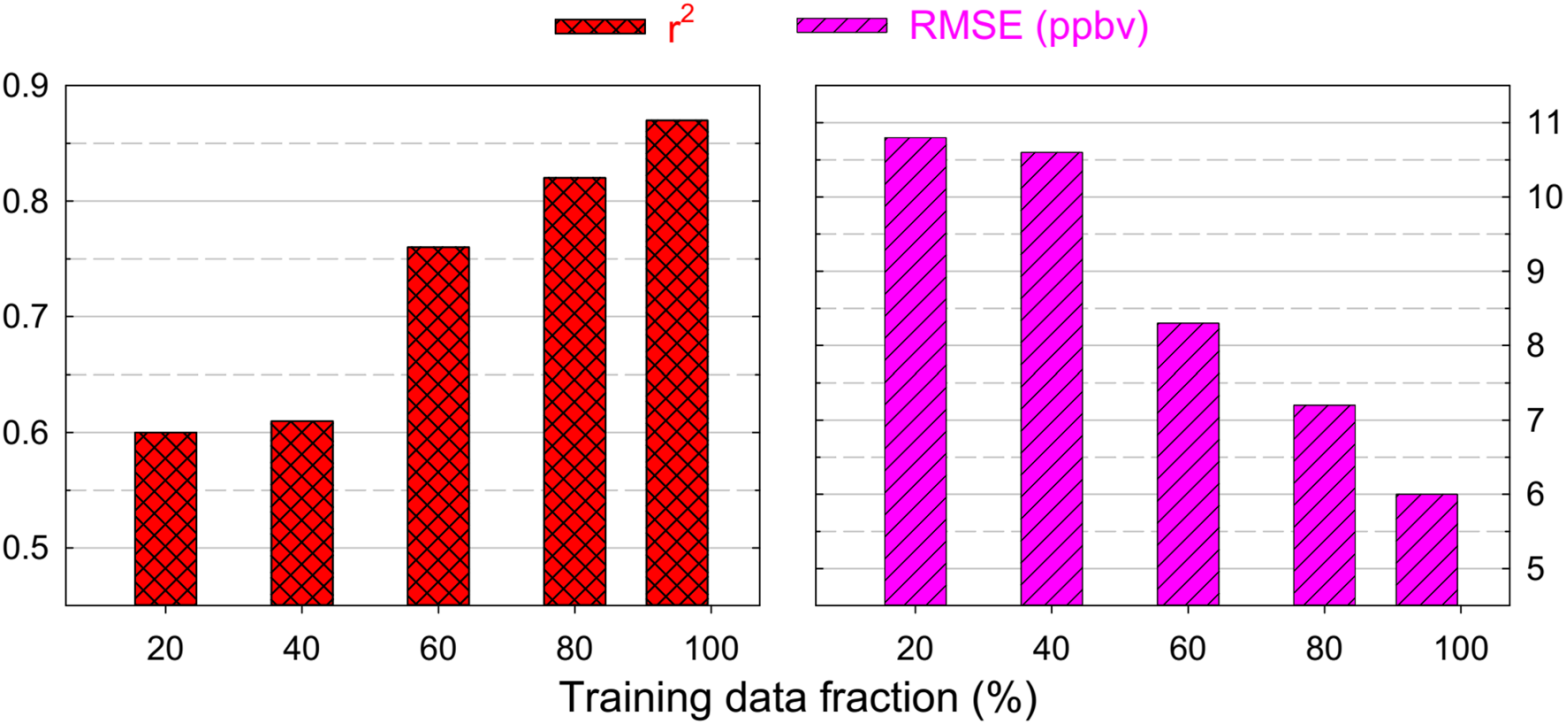
Variation in r^2^ and RMSE with change in the percentage data used in training of ML model.

We further investigate the sensitivity of model performance to the fraction of available data being used for the training. In this regard, a series of simulations have been performed using the best performing model set up (ML_cams_O_3__met_prec) by using 20–95% data for model training. Figure 4 shows the variations in r^2^ and RMSE values due to variation in the training data fraction. The analysis shows that the model performance is highly sensitive to the length of total data being used in its training. The r^2^ value is found to increase significantly from about 0.6–0.87 and RMSE shows reduction from ~ 11 to 6 ppbv with increase in the training data fraction. The analysis suggests that longer time-dependent datasets are highly desirable for optimizing performance of ML models in predicting air quality variation. This underlines that long-term in situ measurements and validated chemistry-climate simulations can help in further exploiting the potential offered by the ML approach.

## Discussion

Our study unravels the strong potential of ML modeling for computationally inexpensive simulations of urban O_3_ variability in the Himalayan foothills region. The periodicity in O_3_ and meteorological parameters due to systematic seasonal cycle of India tends to allow ML model to reproduce data fairly well. In lack of high-resolution measurements, ML simulations can be used to assess the impacts of O_3_ on health and agriculture in this region. Additionally, the series of simulations conducted here would serve as a reference for further applications of AI/ML based modeling to complement conventional Earth system models. It is however pointed out that here the environment is urban and the O_3_ variations are greatly governed by the regional photochemistry. The scenario could be very different for cleaner remote regions where O_3_ variability is dominated by transport from upwind polluted regions or from the higher altitudes. In this regard, we recommend establishing baseline stations to continuously monitor the atmospheric composition as well as the meteorology to exploit the full potential of ML modeling. Model performance is already promising with inclusion of only meteorology, nevertheless, the inclusion of precursors enhances the model’s ability to capture outliers, which are critical in air quality assessments. Future studies may extend the scope to additional climate-forcing pollutants and to unravel feedback between pollution and meteorology causing calamities in the fragile ecosystem of the Himalaya experiencing strong anthropogenic pressure.

## Supplementary Information

Supplementary Information.
