# Supplementary material for: Exploring the potential of machine learning for simulations of urban ozone variability
Source: Sci Rep. 2021 Nov 18;11:22513. doi: 10.1038/s41598-021-01824-z (PMC8602617; doi:10.1038/s41598-021-01824-z)
Supplement: Supplementary file 1 — Supplementary Information. [file 41598_2021_1824_MOESM1_ESM.docx]

Supplementary material

**Exploring the potential of machine learning for simulations of urban ozone variability**

**Narendra Ojha^1*^, Imra­n Girach^2*^, Kiran Sharma^3^, Amit Sharma^4^, Narendra Singh^5^, and Sachin S. Gunthe^6, 7^**

^1^Physical Research Laboratory, Ahmedabad, India

^2^Space Physics Laboratory, Vikram Sarabhai Space Centre, Thiruvananthapuram, India

^3^Graphic Era (deemed to be University), Dehradun, India

^4^Department of Civil and Infrastructure Engineering, Indian Institute of Technology Jodhpur, Jodhpur, India

^5^Aryabhatta Research Institute of Observational Sciences, Nainital, India

^6^EWRE Division, Department of Civil Engineering, Indian Institute of Technology Madras, Chennai, India

^7^Laboratory for Atmospheric and Climate Sciences, Indian Institute of Technology Madras, Chennai, India

***Correspondence:** N. Ojha ([ojha@prl.res.in](mailto:ojha@prl.res.in)) and I. Girach ([imran.girach@gmail.com](mailto:imran.girach@gmail.com))

For submission to Scientific Reports (August 2021)

This supplementary material contains 1 Table.

**Table S1:** Hyper parameters used in the ML model for simulating urban O_3_ variations

| Parameters | Set values | |
| --- | --- | --- |
|  | For ML_obs_O_3__met_prec | For all other simulations |
| booster (type of boosting method to be used) | Gbtree | Gbtree |
| colsample_bylevel (subsample ratio of columns when constructing each level) | 1 | 1 |
| colsample_bynode (subsample ratio of columns for each node (split)) | 1 | 1 |
| colsample_bytree (subsample ratio of columns when constructing each tree) | 0.5 | 0.9 |
| gamma (Minimum loss reduction required to make a further partition on a leaf node of the tree) | 400 | 0.3 |
| learning_rate or eta (step size shrinkage used in update) | 0.04 | 0.05 |
| max_delta_step (Maximum delta step we allow each tree’s weight estimation to be) | 0 | 0 |
| max_depth (Maximum depth of a tree) | 3 | 4 |
| min_child_weight (Minimum sum of instance weight needed in a child) | 3 | 13 |
| n_estimators (Number of gradient boosted trees. Equivalent to number of boosting rounds) | 2000 | 2000 |
| n_jobs (Number of parallel threads used to run xgboost) | 1 | 1 |
| nthread (Number of parallel threads used to run XGBoost) | None | None |
| Objective (specify the learning task and the corresponding learning objective function to be used) | reg:squarederror | reg:squarederror |
| random_state (random number seed) | 0 | 0 |
| reg_alpha (L1 regularization term on weights) | 1.6 | 100 |
| reg_lambda (L2 regularization term on weights) | 0.05 | 0.05 |
| scale_pos_weight (balancing of positive and negative weights) | 1 | 1e-15 |
| seed (random number seed) | None | 500 |
| Subsample (subsample ratio of the training instances) | 0.6 | 0.9 |
| base_score (initial prediction score) | 1.1 | 1 |
